# Part I of Finnish Agility Dog Survey: Training and Management of Competition-Level Agility Dogs

**DOI:** 10.3390/ani12020212

**Published:** 2022-01-17

**Authors:** Leena Inkilä, Heli K. Hyytiäinen, Anna Hielm-Björkman, Jouni Junnila, Anna Bergh, Anna Boström

**Affiliations:** 1 Department of Equine and Small Animal Medicine, Faculty of Veterinary Medicine, University of Helsinki, FI-00014 Helsinki, Finland; heli.hyytiainen@helsinki.fi (H.K.H.); anna.hielm-bjorkman@helsinki.fi (A.H.-B.); anna.bostrom@helsinki.fi (A.B.); 2 EstiMates Ltd., Kamreerintie 8, FI-02770 Espoo, Finland; jouni.junnila@estimates.fi; 3 Department of Clinical Sciences, Swedish University of Agricultural Sciences, 75007 Uppsala, Sweden; anna.bergh@slu.se

**Keywords:** dog agility, canine sports medicine, agility training, dog training, training routines, training practices, competition routines, management of sporting dogs, dog sports

## Abstract

**Simple Summary:**

Information on training, competition, and management of agility dogs is sparse. To decrease this knowledge gap, Finnish owners and handlers of competition-level agility dogs completed an online questionnaire to describe the agility routines of their dog during one injury-free year. Additional information on competition routines was retrieved from the national competition results database. Typically, competition-level agility dogs trained agility once or twice a week and competed two runs a month. The median total weekly training time was 18 min. Usual speed over the competition course was 4.3 m/s. Artificial turf, with or without filling, and dirt surfaces are used most often. Dogs are warmed up before and cooled down after agility performance. Most dogs visit a massage therapist, physiotherapist, osteopath or other professionals of musculoskeletal care at least every three months. Many dogs undergo conditioning exercises, although often less often than every two weeks. Additionally, agility dogs are walked for a total of 1.5 h a day. Dogs competing at the highest levels competed more but trained less than dogs at lower levels. This is the first investigation of agility-related routines in competing agility dogs.

**Abstract:**

Knowledge regarding training, competition, and management routines of agility dogs is lacking. Through a retrospective online questionnaire, Finnish owners and handlers of 745 competition-level agility dogs provided information on training routines and management of these dogs during one year free of agility-related injuries. Competition routines were collected from the national competition results database. Most dogs trained agility 1–2 times a week, with a median active training time of 18 min a week. Dogs competed in a median of 2.1 runs per month at a speed of 4.3 m/s. Common field surfaces were different types of artificial turfs and dirt surface. Warm-up and cool-down were established routines, and 62% of dogs received regular musculoskeletal care. Moreover, 77% of dogs underwent conditioning exercises, but their frequency was often low. Additionally, dogs were walked for a median of 1.5 h daily. Pearson’s chi-squared and Kruskal–Wallis tests were used to evaluate the association between a dog’s competition level and training and competition variables. A dog’s competition level was associated with competition (*p* < 0.001) and training frequency (*p* < 0.001); dogs at higher levels compete more but train less than dogs at lower levels. This study provides information on training, competition, and management routines of competing agility dogs.

## 1. Introduction

Dog agility is a growing sport worldwide, with a significant risk for sport-related injury in dogs [1,2]. In agility, dogs are guided through an obstacle course as quickly as possible by their handlers. Each performance is called a “run”, and in competitions dogs often perform multiple runs per day—in Finland in general up to three. Dogs compete at three levels, classes 1 to 3, with the top dogs of class 3 qualifying for national championships or the national team. In some European countries, including Finland, dogs are divided into five height categories by their height at withers with different fence heights in each category [3]. In several international events, such as the Fédération Cynologique Internationale (FCI) World Championships, only three height categories are currently used. In Finland, training and competitions during the winter season, from approximately October to April, take place indoors due to challenging weather conditions, which may result in different training frequencies or surfaces used than during the summer season, when outdoor facilities can also be used.

Information on training and competition routines and management of agility dogs is sparse. This type of information is typically attained through surveys directed to dog owners and handlers [4,5], but competition databases could also be utilised, as with racing greyhounds [6]. In North American populations, agility dogs train on average two to three times a week and for most dogs the total training time is up to two hours a week [4,5]. The number of competition events per year is on average 20 [5], but the number of runs per event or year is unknown. 

Reports on training routines in other canine sports are also sparse. Typical training of greyhounds consists of two high-intensity workouts or races a week with low-intensity work, such as walking, trotting or free exercise, in between [7]. Warm-up, cool-down, and conditioning exercises are carried out by most agility dogs [1,4], which differs from greyhounds who perform warm-up infrequently [8]. Musculoskeletal care, with the aim of preventing or treating injuries or improving performance, is provided to 40–60% of agility dogs, particularly to those with agility-related injury [4,9]. Massage, chiropractic care, and acupuncture have been utilised [4,9].

Nevertheless, documentation of many sport-related details, such as duration of training sessions, competition speed of dogs, field surfaces, fence heights in training, and content of warm-up and cool-down, is lacking. In addition, observations from the sport suggest that the sport has developed in recent years, with increasing competition speeds and a higher number of agility training facilities. These changes may have affected the training and management routines over the years. 

This is the first part of the Finnish Agility Dog Survey. The overall aim of this first part of the study was to provide knowledge regarding training, competition and management of agility dogs. A specific aim was to describe training and competition routines of competing agility dogs using survey data provided by dog owners and handlers as well as competition results from the national competition database. Additionally, we aimed to report the management routines, such as exercise and musculoskeletal care, of competition-level agility dogs. Finally, we aimed to evaluate how routines and performance differ across height categories and competition levels.

## 2. Materials and Methods

### 2.1. Dogs and Respondents

Owners and handlers of competition-level Finnish agility dogs actively participating in the sport were invited to take part in an online survey. Respondents gave their consent by participating in the survey. Their dog had to have competed in agility in Finland in 2018 and/or 2019 and trained agility during 2019. One questionnaire was completed per dog, identified by registration number, and one respondent was allowed to complete the questionnaire of multiple dogs. If the survey was completed twice for one dog, the first given answers were used. The link to the online questionnaire was distributed via the Finnish Agility Association and social media (multiple Facebook pages) to Finnish agility handlers from July to September 2020. 

The questionnaire was used to collect information for two different studies (Part I and II). Therefore, some questions relate to injuries during 2019. These injury-related results are reported in Part II of the article [10]. Thus, dogs with agility-related injuries during 2019 were excluded from Part I of this study.

### 2.2. Questionnaire

Development of the retrospective online questionnaire utilised expert opinions from veterinarians, veterinary physiotherapists, a statistician, a researcher experienced in survey studies, and experienced agility judges and competitors. The questionnaire was further tested using the cognitive interview method [11], a test group and a checklist concerning things to ensure before publishing a survey [12]. The final questionnaire (S1) consisted mainly of closed-end multiple-choice questions in the Finnish language. Most questions were compulsory but included an escape option such as “I don’t know” or “I can’t remember”. The respondent had the opportunity to provide details in open-field boxes, if needed. The questionnaire utilised skip logic; the following questions appeared or not, depending on the responses to the previous questions. Thus, only relevant questions were shown to each respondent, resulting in a varying total number of questions across respondents. The questionnaire was not anonymous because of the possible need to re-contact for inconsistencies in answers.

The questionnaire included questions about signalment, dog’s and handler’s experience in agility, training routines during 2019, musculoskeletal care and exercise routines during 2019, and the health history of the dog. Table 1 and Table 2 show the details of background information and the information regarding training, competition, and management collected, respectively.

### 2.3. Competition Results Database

The Finnish Agility Association holds a database for all national competition results. Competition-related variables, listed in Table 2, were retrieved from this database using the individual dog’s registration number and were combined with the survey answers of each dog. In the database, a dog’s competition speed is calculated using a length measurement of its expected route for a specific course and its course time.

### 2.4. Data Curation

Inconsistent answers, such as veterinary diagnoses and open-field descriptions not in agreement with each other, were corrected according to the open-field answers. If the open-field answer was inconclusive, the answer was considered missing. 

Health history information on carpal sprains and sprain of a toe was retrieved from open-field descriptions of “other ligament injury” because they were common among the open-field answers. If the date of diagnosis of patellar luxation, osteochondrosis/osteochondritis dissecans (OC[D]), injury of the biceps tendon or muscle, injury of the supraspinatus muscle or tendon, shoulder instability/medial shoulder syndrome, fracture, other muscle injury, carpal sprain or sprain of a toe was not available, the information was considered missing. Only diagnoses made prior to 2019 were included to ensure that the dog had been able to participate in agility after the diagnosis. Thus, these are diagnoses that did not result in retirement from the sport in these dogs.

For comparison of training-related routines across competition levels, two variables were categorised as follows: frequency of training sessions (<2, 2, >2 sessions/week) and training session duration (up to 10 min, 10–15 min, at least 15 min). The variable categorised training session duration was additionally used for analysing differences between seasons. To analyse the effect of season on main field surface, the field surface variable was categorised as follows: dirt/sand, artificial turf without filling, artificial turf with rubber filling, artificial turf with cork filling or other.

Maximum relative fence height in competitions was calculated using the upper range for fence height of each height category according to national competition regulations. According to the national competition regulations, ‘Extra Small’ dogs are allowed to compete in the ‘Small’ category and ‘Small Large’ dogs in the ‘Large’ category if the handler desires. Therefore, we asked about the typical competition category of these dogs, and the maximum fence height of that category was used in the calculation of the maximum relative fence height in competitions.

Weekly total training time was estimated using frequency of training sessions, training session duration, and duration of breaks. Weekly total training time was calculated for active training weeks, excluding weeks off agility. The value for weekly total training time combined training frequencies and durations, which were reported separately for winter and summer. This value was balanced using the number of active training weeks during each season. If the frequency of the training session was reported as less than once a week, an estimation of training every other week was used. For frequency of over 20 times a week, estimation of 21 sessions/week was used. If training session duration was reported as 5–10 min, the value of 7.5 min/session was used. Similarly, the middle value of each range was used for other ranges (10–15 min, etc.). An estimation of 2.5 min/session was used if training session duration was reported as below 5 min, and 32.5 min/session if reported session duration was above 30 min.

Weekly active agility time was estimated by combining weekly total training time and competition frequency. Each competition run was estimated to have one minute of active participation in agility, and total number of runs during the year 2019 was divided by the number of active training weeks. 

“I don’t know” or “I can’t remember” answers in multiple-choice questions were handled as missing values. This, together with skip logic, led to a different total number of answers for many variables. 

### 2.5. Data Analysis and Statistical Methods 

A total of 6431 dogs with Finnish registration numbers had competed in Finland during 2018 and/or 2019. The exact number of dogs fulfilling our criterion of having additionally trained agility during 2019 is unknown; thus, our target population is estimated to be between 5500 and 6000 individual dogs. The final sample size of this study was, however, dictated by the number of responses received during data collection. 

Descriptive statistics (median, interquartile range, frequency table) were calculated for all variables. Normality of the data was tested using the Shapiro–Wilk test. Because of non-normality, Pearson’s chi-squared test or the Kruskal–Wallis test with Bonferroni corrections for the pairwise comparisons were used to evaluate the association of height category with the following variables: relative fence height in training, performance technique on the A-frame and dogwalk, competition frequency, and proportion of faultless runs. The same tests were used to analyse the association of a dog’s highest competition level with the following variables: competition and training frequency, training session duration, weekly active agility time, relative fence height in training, competition speed, proportion of faultless runs, performance technique on A-frame and dogwalk, regular musculoskeletal care, conditioning, and participation in other physically demanding sports. Cramer’s V value was used to report the effect size of Pearson’s chi-squared tests. Z-test with Bonferroni corrections was utilised to evaluate differences between categories. Difference between relative fence height in training and maximum relative fence height in competitions was analysed with the Sign test because the difference was asymmetrical. In addition, Spearman’s correlation coefficient was calculated for dog’s competition speed and proportion of faultless runs.

The difference between summer and winter seasons in the frequency of training sessions and categorised training session duration was analysed with subject-specific cumulative logit-models for repeated measures using the season (winter/summer) as the sole fixed factor and dog as the random subject effect. The Kenward–Rogers method was used in calculating degrees of freedom and adjusting for standard errors with fixed effects. The Newton–Raphson technique was used as the optimisation method. The difference in the variable of categorised main field surface between summer and winter seasons was analysed with a subject-specific multinomial logit-model with season as the sole fixed factor and dog as the random subject effect. Odds ratios (ORs) with 95% confidence intervals (CIs) were calculated from the models. 

Significance was set at *p* < 0.05. Statistical analyses were performed using SPSS (version 26, IBM Corp, Armonk, NY, USA) and SAS (version 9.4, SAS Institute Inc., Cary, NC, USA).

## 3. Results

Continuous variables are presented as median (interquartile range). 

### 3.1. Dogs and Respondents

**Figure 1:**
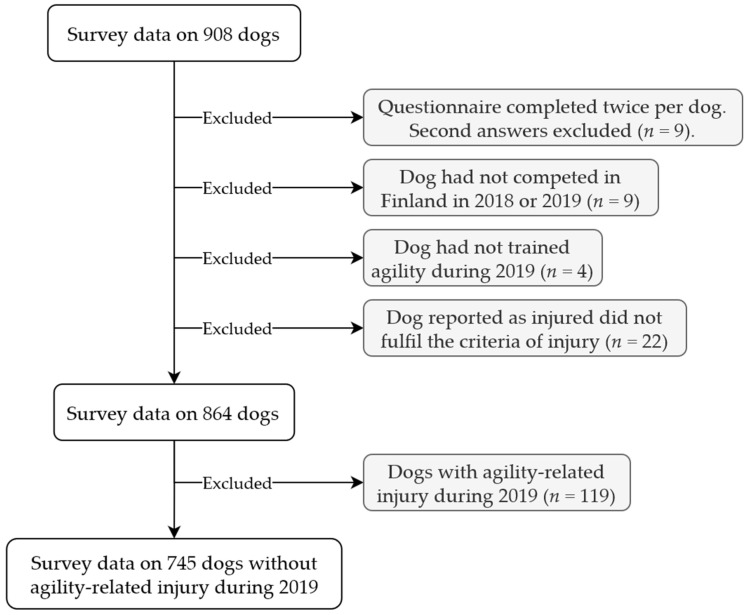
Dogs included in this study.

Survey data from 670 individual respondents concerning 745 dogs without agility-related injury during 2019 were used in this study (Figure 1).

In the group of 745 dogs, the age of the dogs at the beginning of 2019 was 4.4 years (2.8–6.1 years). Weight and height were 13.0 kg (8.0–18.5 kg) and 42.0 cm (34.0–50.0 cm), respectively. Dogs represented the following height categories: Extra Small (7.4%), Small (20.3%), Medium (24.2%), Small Large (21.7%), and Large (26.4%).

Further, 33.4% were intact females, 27.2% spayed females, 28.3% intact males, and 11.0% neutered males. Eighty-eight different breeds were presented, with the most popular ones being Border Collie (16.1%), Shetland Sheepdog (11.1%), Australian Shepherd (5.4%), Spanish Water Dog (5.4%), Belgian Shepherd (4.3%), Parson Russell Terrier (3.6%), Australian Kelpie (3.5%), Jack Russell Terrier (3.4%), and Collie (2.8%). Table S1 provides a list of all breeds.

#### Dog’s and Main Handler’s Experience in Agility

**Figure 2:**
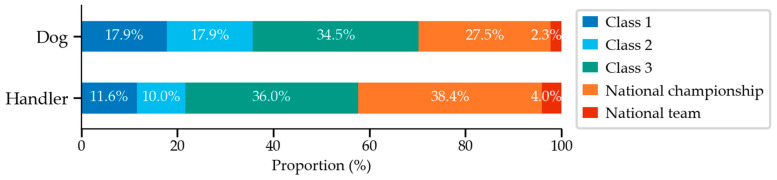
Dogs’ (*n =* 745) and main handers’ (*n =* 670) highest competition level.

Age for starting course-like practice (sequences of at least 5 obstacles) was 1.0 years (0.8–1.2 years, *n =* 735). Jumps were set at competition height at the age of 1.5 years (1.3–2.0 years, *n =* 711). Dogs had started competing in agility at the age of 2.2 years (1.8–3.0 years, *n =* 741). At the end of 2019, the length of the competition career was 2.7 years (1.4–4.5 years, *n =* 741). Main handlers had 9.0 years of experience in agility (5.0–14.0 years, *n =* 670). Figure 2 shows dogs’ and main handlers’ highest competition levels. 

### 3.2. Training, Competition, and Management Routines during 2019

#### 3.2.1. Agility Training

**Figure 3:**
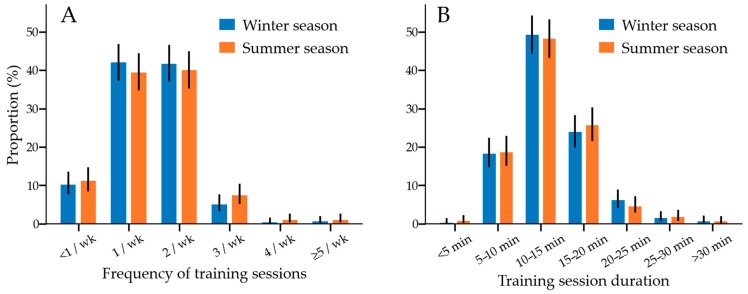
(**A**) Frequency of training sessions during winter (*n =* 734) and summer (*n =* 703) seasons. (**B**) Training session duration during winter (*n =* 733) and summer (*n =* 702) seasons. Error bars are used to indicate 95% confidence intervals. The frequency of training sessions or training session durations did not significantly differ between the winter and summer seasons.

The number of weekly agility training sessions ranged from less than 1 to more than 20 in both winter and summer seasons. Most dogs trained once or twice a week for 5–20 min (Figure 3). 

Weekly total training time was 18 min/week (13–25 min/week; *n =* 730). Only 0.5% of dogs trained for more than two hours a week. A dog’s highest competition level was associated with weekly total training time (*p* < 0.001, *n =* 730); dogs that had been part of the national team trained a median of 10 min/week, which was less than class 1 dogs (*p* = 0.001; median 25 min/week), class 2 dogs (*p* = 0.012; median 19 min/week), and dogs that had participated in national championships (*p* = 0.043; median 18 min/week). Additionally, class 3 dogs, training a median of 18 min/week, trained less than class 1 dogs (*p* = 0.008). 

**Figure 4:**
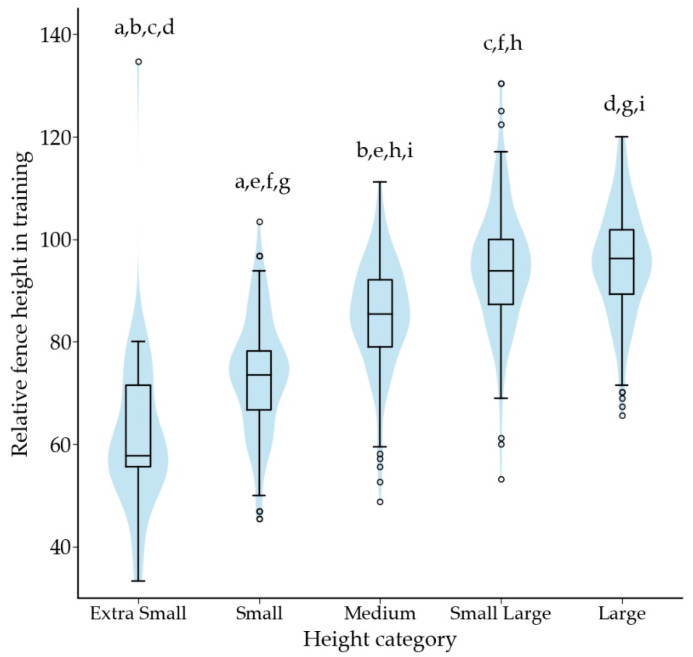
Box plot showing relative fence height in training according to the height category. Same letter indicates a significant difference between two height categories. For letter a, *p* < 0.05, and for letters b to i *p* < 0.001. Outliers are plotted as “◦”.

The relative fence height in training was 88% (76–96%, *n =* 744) of the dog’s height at withers. The height category of the dog had a significant association with relative fence height (*p* < 0.001); relative fence height increased with the height of the dog (Figure 4). A dog’s highest competition level was associated with its relative jump height in training (*p* = 0.001), with class 1 dogs (median 83%) jumping significantly lower fences than dogs that had participated in national championships (*p* = 0.002; median 91%) or been part of the national team (*p* = 0.050; median 90%).

**Figure 5:**
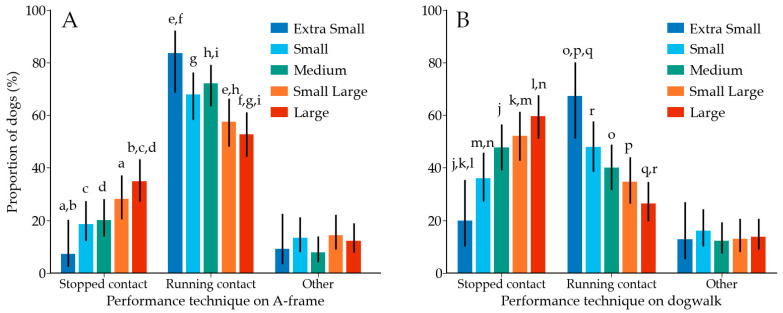
(**A**) Performance technique on A-frame across height categories of dogs. Proportion of dogs within each height category is shown. (**B**) Performance technique on dogwalk across height categories. Same letter indicates a significant difference (*p* < 0.05) between two height categories. Error bars are used to indicate 95% confidence intervals.

A-frame was performed with the following performance techniques: running contact (63.9%; 472/739), stopped contact (24.5%; 181/739) or other/in between (11.6%; 86/739). Dogwalk was performed with the following performance techniques: stopped contact (47.4%; 352/742), running contact (38.9%; 289/742) or other/in between (13.6%; 101/742). Height category, but not the dog’s highest competition level, was associated with performance technique on A-frame (*p* < 0.001; effect size 0.152) and dogwalk (*p* < 0.001; effect size 0.172) (Figure 5).

#### 3.2.2. Competition

**Figure 6:**
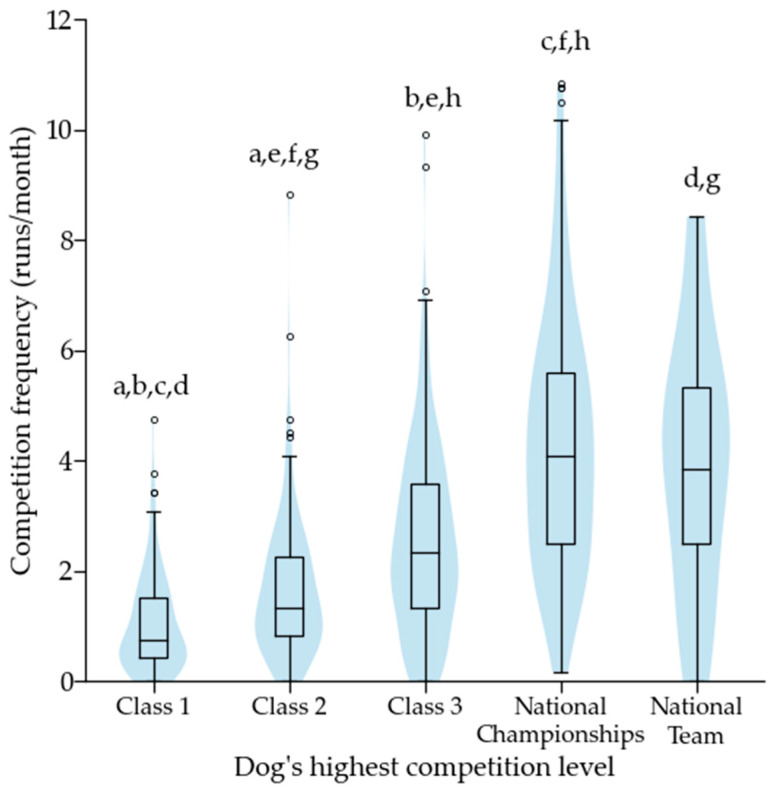
Average number of monthly competition runs during 2019 according to a dog’s highest competition level. Same letter indicates a significant difference between two height categories. For letter a, *p* < 0.01, and for letters b to h *p* < 0.001. Outliers are plotted as “◦”.

**Figure 7:**
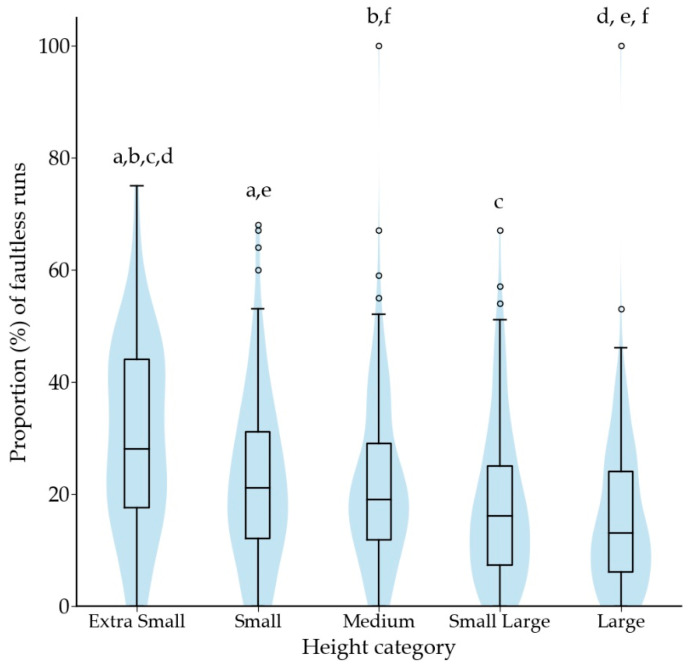
Proportion of faultless runs in 2018 and 2019 according to the height category. Same letter indicates significant difference between two height categories. For letters a and b, *p* < 0.05, and for letters c to f *p* < 0.001. Outliers are plotted as “◦”.

During 2019 the dogs competed a median of 2.1 competition runs per month (1.0–3.8 runs per month; *n =* 745). The average number of monthly competition runs differed across a dog’s highest competition level (*p* < 0.001), with competition frequency increasing with competition level (Figure 6). Competition frequency did not differ across height categories. The proportion of faultless runs was 17% (9–28%, *n =* 745). The proportion of faultless runs increased with the dog’s highest competition level (*p* < 0.001); all levels significantly differed from each other (*p* < 0.005), except that class 2 did not differ from class 3, and national championship participants did not differ from national team members. The proportion of faultless runs differed across the height categories (*p* < 0.001), with a higher proportion in the smaller height categories (Figure 7).

**Figure 8:**
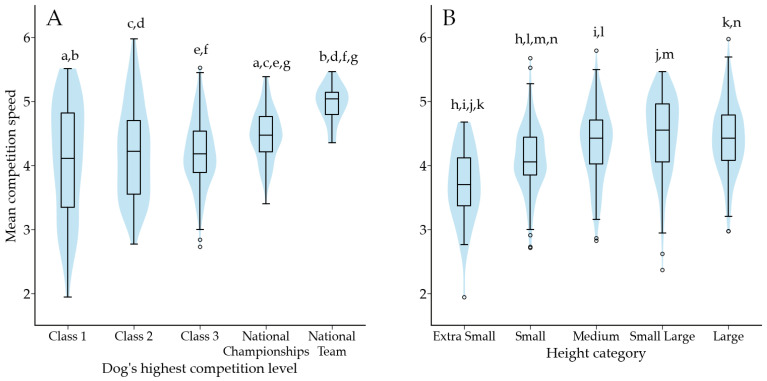
(**A**) Mean speed of faultless runs according to the dog’s highest competition level. (**B**) Mean competition speed according to the height category. Same letter indicates a significant difference between two competition levels or height categories. For letter a, *p* < 0.05, for letters g, h and l *p* < 0.01, for all other letters *p* < 0.001. Outliers are plotted as “◦”.

Competition speed was 4.3 m/s (3.9–4.7 m/s; *n =* 669) across all levels. Competition speed was associated with a dog’s highest competition level (*p* < 0.001) and height category (*p* < 0.001) (Figure 8). The proportion of faultless runs had a weak negative correlation with competition speed (r_s_ = −0.199; *p* < 0.001; *n =* 669). 

**Figure 9:**
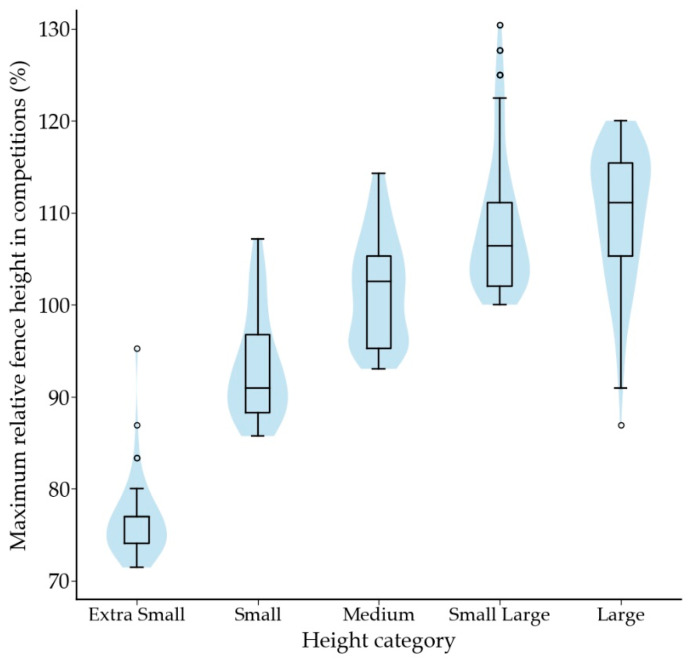
Maximum relative fence height in competitions according to the height category. Significant difference (*p* < 0.001) was observed between all height categories, except between Small Large and Large. Outliers are plotted as “◦”.

Maximum relative fence height in competitions was 103% (95–109%; *n =* 745). Maximum relative fence height in competitions increased with height category (*p* < 0.001) (Figure 9). Usual relative fence height in training differed from maximum relative fence height in competitions (*p* < 0.001; *n =* 744): for 86.4% of dogs the usual fence height in training was below the maximum fence height used in competitions.

Amount of weekly agility, combining training and competitions, was 19 min (13–27 min, *n =* 729) during the weeks that the dog participated in agility. 

#### 3.2.3. Field Surface

Table 3 shows the main surfaces used in agility training and competitions. The main field surface significantly differed between the winter and summer seasons (*p* < 0.0001). In the summer season, the proportion of dogs using dirt or sand (OR 8.32, CI 6.10–11.35) or other surfaces (grouped value including natural grass, artificial turf with sand filling, horse-riding surfaces, fibre-sand mix or rubber mat) (OR 1.83, CI 1.15–2.91) as main surfaces compared to artificial turf with rubber filling, were increased compared to the same proportions in the winter season.

#### 3.2.4. Time off from Agility

Some dogs (7.1%; 53/745) trained agility only during winter or summer. During the winter season, 52.3% (384/734) of dogs had time off from agility, with similar numbers (47.2%; 332/703) for the summer season. For dogs that had had time off, the total duration of breaks was 5 weeks (4–8 weeks; *n =* 371) in the winter season and 4 weeks (3–5 weeks; *n =* 327) in the summer season. When considering all dogs that had trained during both seasons, the total duration of time off from agility during the year was 4 weeks (0–8 weeks; *n =* 678). One-third (35.43%; 239/678) of dogs had no breaks from agility during 2019. Table 4 presents the reasons for breaks during the winter and summer seasons. Multiple reasons were chosen for 19.3% (74/384) of dogs in the winter season and for 18.7% (62/332) of dogs in the summer season.

#### 3.2.5. Warm-Up and Cool-Down

A warm-up before agility training and competition runs was performed either always (93.4%; 696/745), usually (6.0%; 45/745), sometimes (0.4%; 3/745), or never (0.1%; 1/745). Warm-up durations for 743 dogs were <5 min (0.4%), 5–10 min (10.5%), 10–15 min (31.2%), 15–20 min (33.6%), 20–25 min (11.8%), 25–30 min (10.5%), and >30 min (1.9%).

A cool-down was performed either always (86.7%; 646/745), usually (12.2%; 91/745), sometimes (0.9%; 7/745), or never (0.1%; 1/745). Cool-down durations for 744 dogs were <5 min (1.2%), 5–10 min (13.0%), 10–15 min (26.9%), 15–20 min (28.8%), 20–25 min (15.9%), 25–30 min (10.3%), and >30 min (3.9%).

Table 5 shows the elements of usual warm-up and cool-down. Respondents were allowed to select multiple items. The number of chosen items for the warm-up ranged from 1 to 11, with multiple items chosen in 98.1% (730/744) of dogs. For elements of cool-down, the number of chosen items ranged from 1 to 7, with multiple items chosen in 88.0% (655/744) of dogs.

#### 3.2.6. Musculoskeletal Care and Conditioning

The frequency of visits to professionals for musculoskeletal care is presented in Table 6. Most dogs (62.4%; 465/745) received regular musculoskeletal care (at least once every three months), whereas 9.8% (73/745) of dogs did not receive any musculoskeletal care during 2019. The highest competition level was associated with regular musculoskeletal care (*p* < 0.001, effect size 0.208, *n =* 745); a higher proportion of dogs that participated in the national championship (74.1%) or had been part of the national team (94.1%) received regular musculoskeletal care than dogs in class 1 (50.4%) or class 2 (53.4%). 

Conditioning exercises were performed by 76.8% (572/745) of dogs. A dog’s highest competition level was not associated with conditioning. Frequency of conditioning was at least two times a week (17.7%; 101/572), once a week to every two weeks (35.3%; 202/572) or less often than every two weeks (47.0%; 269/572). Conditioning exercises were most often planned by the owner or handler (70.8%; 405/572), followed by the physiotherapist (22.4%; 128/572) and another person (6.8%; 39/572).

#### 3.2.7. Daily Exercise

The total duration of usual daily walks was 1.5 h (1.3–2.0 h; *n =* 717). During walks 6.6% (49/745) of dogs were always off leash, 44.0% (328/745) mostly off leash, 45.6% (340/745) mostly on leash, and 3.8% (28/745) always on leash. About one-fourth (24.2%, 180/745) of the dogs participated in addition to agility in other physically demanding activities such as canicross, herding or hunting. A dog’s highest competition level was associated with participation in other physically demanding activities (*p* = 0.001, effect size 0.16), with a higher proportion of class 1 dogs (33.8%) participating in other activities than dogs that had taken part in national championships (17.6%) or been part of the national team (0.0%).

### 3.3. Health History

Of this canine sample, 22.1% (159/719) had a history of agility-related injury prior to 2019. Among these dogs, the number of agility-related injuries was a median of 1.0 (1–2; *n =* 159). One-fourth (25.7%; 187/729) of dogs had had a non-agility-related musculoskeletal injury during their lifetime.

Table 7 shows the frequency of selected musculoskeletal diagnoses regardless of their aetiology. Diagnoses of hip dysplasia, elbow disease, and lumbosacral transitional vertebra (LTV) were included regardless of the date of diagnosis, as they were considered congenital. Other diseases were included if they had been diagnosed before 2019 to ensure that the dog had participated in agility after the diagnosis. Diagnoses of intervertebral disc disease or injury of the supraspinatus muscle or tendon were not reported by the respondents. Grade of LTV was available for 87 dogs. Most dogs had LTV1 (65.5%; separation of the first spinous process from the median crest of the sacrum or other mildly abnormal structure), with LTV2 (symmetrical LTV) in 3.4% of dogs, LTV3 (asymmetrical LTV) in 18.4% and LTV4 (6 or 8 lumbar vertebrae) in 12.6%.

## 4. Discussion

This study provides detailed information on training, competition, and management routines of Finnish competition-level agility dogs during a period without agility-related injuries. Agility dogs, starting course training generally at one year of age, typically trained agility once or twice a week and competed two runs a month, with one month off each year from agility. Warm-up and cool-down were established routines in the sport, but conditioning exercises were generally performed only occasionally or not at all. However, the total duration of daily walks was high, providing general conditioning. Regular musculoskeletal care was provided for most dogs. Performance and training varied across competition levels and height categories. 

In our sample, agility dogs started course-like training, with sequences of jumps, typically around one year of age, which is similar to greyhounds [7]. According to recommendations, concussive training should not commence before the growth plates of the dog are closed, which is beyond one year, especially in larger dogs [17]. Repetitive training of jumps at a young age has been speculated to potentially increase the risk of injuries at an older age [17]. To our knowledge, no studies have been conducted on the subject—veterinary sports medicine literature on training and management recommendations for agility dogs is sparse overall and mainly based on research in other species or disciplines.

Over 90% of Finnish agility dogs trained agility up to twice a week, which is less than the previously reported average of training 2.5 times per week in the USA [5]. In an American population, over 10% of agility dogs train more than two hours per week [4], whereas in our sample almost no dogs trained that much, with the majority training less than half an hour per week. The marked differences in the amount of training may arise from our team asking only about the active time that the dog spent performing or was rewarded, excluding time spent waiting, warming up or obtaining instructions from the coach, and it is unknown what definition has been applied previously [4]. Additionally, some handlers may have limited access to training facilities in Finland, whereas the availability may be better in the USA. 

The opposite was noted regarding the amount of time that the dogs were walked; over three-quarters of American agility dogs are walked no more than two hours a week [4], whereas in our Finnish population the duration of walks was markedly more, a median of 1.5 h a day. Thus, in the USA, agility training represented a much higher proportion of the physical activity for each dog than in the Finnish population, which may be due to cultural differences in dog walking routines. Daily walks are likely to provide aerobic exercise, which is recommended for agility dogs, although the sport requires predominantly strength and not endurance [17]. Additionally, for some dogs the daily walks may incorporate strength training, such as hiking on varied terrain or running uphill, especially as most Finnish agility dogs are walked at least partly off leash [17]. Thus, the regular daily exercise of Finnish agility dogs may prepare the dogs for the requirements of the sport and possibly decrease the risk of injury.

Differences in training, competition, and management routines were detected across competition levels, which have not been reported previously. High-level agility dogs spent less time training agility on a weekly basis than dogs at lower levels. They had possibly already established the required skill level, which only had to be maintained via minimal training, whereas dogs at lower levels required more training to learn new skills. As previously reported [5], high-level dogs competed more frequently than dogs at lower levels, suggesting that through the career the proportion of competing increases in relation to training.

Previously not reported, dogs competing in class 1 jumped lower relative fence heights in training than dogs competing at higher levels. It appears that dogs are introduced to maximum competition fence heights gradually. The lower end of competition fence height range is possibly used by judges in class 1 competitions, enabling this approach. As dogs at higher competition levels jump higher relative fence heights in training, they are required to alter their kinematics; the increased shoulder flexion over the jump and the increased extension of the lumbar spine and the neck in landing may result in increased stress on the musculoskeletal tissues of dogs at higher competition levels [18]. However, a minority of dogs trained at fence heights at or above the maximum competition range, suggesting that handlers generally choose to train below the maximum requirements of competitions, which may reduce load on the musculoskeletal tissues.

In agreement with previous studies [4,9], warm-up was a common routine among our respondents. Warm-up of agility dogs was more comprehensive than in greyhounds; greyhounds are walked on leash for short distances if at all [8], whereas the agility dogs in our sample were often warmed up for 10–20 min, exercising the dog at various speeds and performing tricks. Exercising the dog before training could be considered as a general warm-up to increase body temperature, respiration rate, and heart rate [19]. Tricks may represent more specific warm-up strategies that could improve the skill and coordination of dogs [19]. Warm-up of agility dogs, consisting of exercise in increasing speeds and tricks, improves the efficiency of musculus triceps brachii [20]—a muscle highly activated during jumping in agility [21]. Based on human studies, neuromuscular warm-up programmes, including exercises to improve strength and balance, decrease the risk of injury [22], suggesting that the warm-up of agility dogs may have beneficial effects. However, more detailed information is needed on the content of tricks to evaluate whether the current warm-up strategies could be more comprehensive. In humans, standard warm-up consisting of aerobic exercise and stretching alone is inferior in reducing injuries than more complete programmes where exercises of strength and balance have been added [23]. Future studies could aim to develop specific warm-up programmes for agility dogs and to test their efficacy in reducing injuries.

Similar to a previous study, three-quarters of the dogs in our study performed conditioning exercises [9]. However, many dogs in our sample performed conditioning exercises infrequently, and the exercises were seldom planned by a professional. Thus, the positive effects that could be gained from structured and regular exercise programmes, such as improved performance or reduced injury risk, are likely to be limited. For example, human athletes perform exercises of injury prevention programmes usually at least twice per week [24,25] and compliance is associated with a protective effect [26]. Conditioning programmes developed specifically for agility dogs by professionals may aid in reducing the risk of sport-related injuries. Future studies could evaluate the effect of professionally planned, sport-specific conditioning exercise plan on dog’s speed or agility performances, and on injury rate.

Musculoskeletal care was used more frequently than previously reported [4,9], with most dogs receiving therapies regularly, at least once every three months. Use of these therapies may have increased as awareness about injury risk has grown. There is no evidence of musculoskeletal care protecting agility dogs from injuries [4,9], but monthly or bimonthly veterinary or physiotherapy evaluations have been recommended for sport dogs [27]. One-third of the competition-level agility dogs were, however, not taken care of by a musculoskeletal care professional on a regular basis, and 10% of dogs did not receive any musculoskeletal care during 2019. This could result in mild injuries going undetected, as lameness or other overt clinical signs may not always be evident [19]. Chiropractors are the most frequently used professionals in the USA [4], whereas in Finland, massage therapists and physiotherapists were consulted most often. Regional differences in availability and education of professionals are likely.

The agility dogs in our study competed at a median of two runs per month; this is less than in greyhounds, which compete at a median of every seven days [6]. In agility, dogs often perform multiple competition runs in the same competition event, resulting in an even lower frequency of actual competition events, but in a higher load per event, which may introduce injury risk due to fatigue. Agility dogs in our study started competing at a median age of 26 months, whereas greyhounds start their racing career younger, at a median age of 21 months [6]. This suggests that acquisition of sufficient skills may take longer for agility than for racing. An alternative explanation may be that agility is usually trained non-professionally as a hobby, which may prolong the training process, whereas greyhound racing is in most countries a professional industry. Additionally, greyhounds are purposely bred for racing, potentially hastening their training process. Some of the agility dogs in our study started the sport at an adult age, whereas greyhounds rarely start racing later than the age of two years [6].

The proportion of faultless runs is greater in dogs competing at higher levels, reflecting their improved skill level despite the increased difficulty of the courses, than in dogs at lower levels. The finding agrees with previous results of higher-level dogs performing obstacles with fewer faults and navigating through the course with greater success [28]. As expected, these high-level dogs were also faster [28], which was confirmed by our study; dogs that had participated in national championships or been part of the national team had significantly higher competition speeds than other dogs. It has been suggested that improved motor control automaticity associated with obstacle performances in high-level dogs allows for greater speed and better detection of the handler’s signals [28]. Supporting this idea, experienced agility dogs use limb dynamics that allow them to re-establish their horizontal speed at landing through increased accelerative impulses compared with beginner dogs [29]. 

Differences across height categories were observed in relative fence heights and performance. Smaller dogs jumped lower relative fence heights in training and competition, which is due to the regulations in the sport. To our knowledge, there are no studies showing that larger dogs have superior jumping abilities to smaller dogs. Although lower relative fence heights have been associated with increased speed [30,31], and smaller dogs had a lower competition speed than larger dogs. Lower speed and lower mass result in lower kinetic energy, and thus, smaller risk of damage in case of collision. Higher gallop speed in larger dogs is associated with increased peak vertical force applied to the trailing hind limb and greater accelerative impulses of both hind limbs [32]. As larger dogs are both faster and jump higher in relation to their height, their musculoskeletal system is under greater stress, which may lead to injuries.

The design of this study entails some limitations. Some owners or handlers may have been more motivated to participate than others resulting in selection bias. Three quarters of the handlers had at least five years of experience in agility, suggesting that experienced handlers may have been overrepresented. Due to social desirability bias, respondents may have chosen answers that they consider as socially preferred [33]. This may have affected our results, such as use of warm-up or amount of training. To reduce this effect, we aimed to formulate all questions neutrally without addressing acceptability to any specific choices [33]. Although not anonymous, the online questionnaire approach may have decreased the bias compared with face-to-face interviews [34]. As the survey was opened more than six months after the end of 2019, some respondents may have had trouble remembering all details. Therefore, we included the response option of “I don’t know/I can’t remember” for almost all questions, allowing respondents to skip questions. In many questions, we asked about usual or general routines such as length or frequency of training sessions. However, these routines may have varied markedly during the season or year, which could not be detected by this questionnaire. Thus, this study provides knowledge of general trends in the sport of agility.

To determine whether respondents understood the questions as intended, cognitive interviews were utilised during the development of the questionnaire. However, it is possible that some respondents approached the questions differently than others. Some respondents may have had difficulties recalling routines during the past year, leading to possible error unless the respondent utilised the “I don’t remember” option. In online questionnaires, it is also possible that respondents accidentally check a box. Therefore, answers were corrected according to the open-field descriptions if the checked box and the description were not in agreement. If checked boxes and open-field descriptions were inconclusive, the answer was considered missing. Despite these issues, the survey approach is justifiable, as it would be very difficult or impossible to obtain much of the information in other ways. This study contributes an overview on the subject, whereas a prospective setup or an activity monitoring could be used to obtain more detailed information. 

## 5. Conclusions

In this Part I of the Finnish Agility Dog Survey, we presented the development and use of the questionnaire on routines of agility dogs. This study provides a base of knowledge of training, competition, and management routines of competition-level agility dogs in Finland during an injury-free year. Across competition levels, there are differences in training and competition routines, speed, competition success and management of the dogs. Height categories differed in relative fence heights in training and competitions, obstacle performance techniques, competition speed, and competition success. This knowledge can be utilised by national and international agility handlers and trainers, veterinarians, physiotherapists, and other professionals that prepare the dogs for the demands of the sport. Part II of the Finnish Agility Dogs Survey describes the dogs with agility-related injuries and risk factors for injuries. 

## Figures and Tables

**Table 1:** Questions regarding demographic and background information.

Category	Variable
Signalment of the dog	Age ^1^ Breed Gender Weight Height Height category ^2^ Weight/height ratio ^1^
Agility experience of the dog	Highest competition level Competition years in agility ^1^ Age at which course-like training started Age at which jumps were set at competition height Age at first competition
Main handler	Years of experience in agility Highest competition level (with any dog)
Health history	Number of previous agility-related injuries ^1^ Non-agility-related musculoskeletal injuries (yes/no) Any of the following musculoskeletal diseases (yes/no): Hip dysplasia (grade C ^3,4^ or worse, or diagnosis by veterinarian)
	Patellar luxation (grade 1 ^3,5^ or worse, or diagnosis by veterinarian) ^8^ Lumbosacral transitional vertebra (LTV; grade 1 ^3,6^ or worse, or diagnosis by veterinarian) Elbow disease (grade 1 ^3,7^ or worse elbow dysplasia, or diagnosis by veterinarian) Osteochondrosis/osteochondritis dissecans (OC[D]; diagnosis by veterinarian) ^8^ Injury of the biceps tendon or muscle (diagnosis by veterinarian) ^8^ Injury of supraspinatus muscle or tendon (diagnosis by veterinarian) ^8^ Shoulder instability/medial shoulder syndrome (diagnosis by veterinarian) ^8^ Fracture (diagnosis by veterinarian) ^8^ Other muscle injury (diagnosis by veterinarian) ^8^ Carpal sprain (diagnosis by veterinarian) ^1,8^ Sprain of a toe (diagnosis by veterinarian) ^1,8^

^1^ Variable was created from the information provided by the survey as part of data curation. ^2^ According to regulations of the Finnish Agility Association: Extra Small (height at withers < 28 cm), Small (28 to <35 cm), Medium (35 to <43 cm), Small Large (43 cm to <50 cm) or Large (≥50 cm). Extra Small dogs are allowed to compete in the Small category and Small Large dogs in the Large category if the handler chooses. ^3^ Health screening result from the Finnish Kennel Club. ^4^ Grading of hip dysplasia from Federation Cynologique Internationale [13]. ^5^ Grading of patellar luxation from the Finnish Kennel Club [14]. ^6^ Grading of LTV from the Finnish Kennel Club [15]. ^7^ Grading for elbow dysplasia according to the International Elbow Working Group [16]. ^8^ Only diagnoses made prior to 2019 were included to ensure that the dog had participated in agility after the diagnosis. The veterinary diagnoses were reported by the respondent.

**Table 2:** Training, competition and management in 2019.

Category	Variable
Training	Frequency of training sessions (average number of sessions per week) ^1^ Training session duration (average active training time per session in minutes) ^1^ Weekly total training time (active training time during active training weeks in minutes, excluding weeks off from agility) ^2^ Main field surface used in training and competitions ^1^ Relative fence height in training (typical fence height in relation to the dog’s height at withers) ^2^ Time off from agility ^1^
Competition ^3^	Competition frequency (average number of competition runs per month) Mean competition speed of faultless runs (m/s) ^4^ Proportion of faultless runs ^4^ Maximum relative fence height in competitions (maximum fence height in relation to the dog’s height at withers) ^2^
Combined training and competition	Performance technique of A-frame and dogwalk ^5^ Weekly active agility time (minutes per week) ^2^
Warm-up and cool-down ^6^	Warm-up (yes/no) Duration of warm-up (average in minutes) Content of typical warm-up Cool-down (yes/no) Duration of cool-down (average in minutes) Content of typical cool-down
Musculoskeletal care	Frequency of visits to physiotherapist, massage therapist, osteopath and other professional Regular musculoskeletal care ^2,7^
Exercise and conditioning	Conditioning (exercises to improve strength, speed, endurance or body control) (yes/no, frequency) ^3^ Duration of daily exercise (average total duration in hours and minutes) Participation in other physically demanding sports (e.g., herding or canicross)

^1^ Asked separately for winter (October to April) and summer (May to September) seasons. ^2^ Variable was created from the information provided by the survey as part of data curation. ^3^ Retrieved from the competition database of the Finnish Agility Association. ^4^ Competition runs from both 2018 and 2019 were used to attain information for as many dogs as possible. ^5^ A-frame and dogwalk can be performed using the following techniques: stopping at the end (stopped contact), running through (running contact) or other/in between. ^6^ In the questionnaire, warm-up and cool-down were defined as exercising the dog before and after agility performance, respectively. The list of elements to tick will be shown in the results section. ^7^ Visit to physiotherapist, massage therapist, osteopath or other professional at least once every three months.

**Table 3:** Main surfaces used in agility training and competitions.

Surface	Winter Season (*n* = 732)	Summer Season (*n* = 702)
Artificial turf with rubber filling	47.8%	27.2%
Dirt or sand	10.7%	45.6%
Artificial turf without filling	21.3%	12.4%
Artificial turf with cork filling	14.3%	8.7%
Artificial turf with sand filling	1.6%	1.6%
Natural grass	0.0%	3.4%
Horse riding surface	1.8%	0.9%
Fibre-sand mix	2.3%	0.1%
Rubber mat	0.1%	0.1%
Other	0.0%	0.1%

**Table 4:** Reasons for time off from agility.

Reason	Winter Season (*n* = 384)	Summer Season (*n* = 332)
Planned break (e.g., periodisation of training)	60.4%	60.2%
Reason unrelated to the dog	28.9%	34.3%
Other dog-related reason	15.4%	14.8%
Previous injury or illness of the dog	9.6%	9.3%
Unknown	0.8%	0.3%

**Table 5:** Elements of usual warm-up and cool-down.

Item	Warm-Up (*n* = 744)	Cool-Down (*n* = 744)
Exercising on leash	92.9%	93.4%
Exercising off leash	58.3%	57.0%
Walking	67.5%	70.8%
Running	72.8%	51.1%
Sprinting	38.6%	2.6%
Tricks	67.2%	5.1%
Active stretches	28.2%	7.0%
Passive stretches	10.2%	5.2%
Tug play	34.3%	3.5%
Habituation to the field surface	30.8%	Not applicable
Obstacle performances as part of warm-up	25.7%	Not applicable
Other ^1^	2.6%	1.9%

^1^ Other elements included, for example, playing with other dogs, massage, or swimming.

**Table 6:** Distribution of treatment frequency of 745 dogs by a massage therapist, physiotherapist, osteopath or other professionals during 2019.

Professional	At Least Oncea Month	Every Two to Three Months	Less Often	Not at all
Massage therapist	11.4%	26.6%	29.0%	33.0%
Physiotherapist	4.8%	19.5%	27.8%	47.9%
Osteopath	0.9%	10.1%	16.5%	72.5%
Other	4.0%	6.6%	5.6%	83.8%

**Table 7:** Frequency of selected musculoskeletal diagnoses in competition-level agility dogs.

Diagnosis	Number of Dogs	Proportion of Dogs
Lumbosacral transitional vertebra	89/745	11.9%
Hip dysplasia	84/745	11.3%
Disease of the elbow	23/745	3.1%
Fracture	21/742	2.8%
Other muscle injury	20/717	2.8%
Patellar luxation	11/744	1.5%
Osteochondrosis/osteochondritis dissecans	8/745	1.1%
Injury of biceps tendon or muscle	5/743	0.7%
Injury of the iliopsoas muscle	4/745	0.5%
Other tendon injury	6/743	0.8%
Cranial cruciate tear	3/745	0.4%
Luxation of the superficial digital flexor tendon	3/745	0.4%
Carpal sprain	2/741	0.3%
Sprain of digit	2/740	0.3%
Osteoarthritis	2/743	0.3%
Spondylosis	2/745	0.3%
Shoulder instability/medial shoulder syndrome	1/745	0.1%

## Data Availability

The data presented in this study are available on request from the corresponding author.

## Supplementary Materials

The following supporting information can be downloaded at: https://www.mdpi.com/article/10.3390/ani12020212/s1, S1: Link to the final questionnaire, Table S1: Breeds of all dogs.
