# Supplementary material for: Part I of Finnish Agility Dog Survey: Training and Management of Competition-Level Agility Dogs
Source: Animals (Basel). 2022 Jan 17;12(2):212. doi: 10.3390/ani12020212 (PMC8772780; doi:10.3390/ani12020212)
Supplement: Supplementary file 1 [file animals-12-00212-s001.zip › animals-1517223-supplementary.pdf]

animals

MDPI

Supplement S1. Link to the final questionnaire: https://elomake.helsinki.fi/lomakkeet/105548/lomake.html

Table S1. Breeds of all dogs.

Breed	Proportion of dogs (n=745)
Border Collie	16.1%
Shetland Sheepdog	11.1%
Australian Shepherd	5.4%
Spanish Water Dog	5.4%
Belgian Shepherd	4.3%
Parson Russell Terrier	3.6%
Australian Kelpie	3.5%
Jack Russell Terrier	3.4%
Collie	2.8%
Border Terrier	2.0%
Mixed breed	2.0%
Medium- sized Poodle	1.9%
Labrador Retriever	1.9%
Miniature Pinscher	1.7%
Miniature Poodle	1.6%
Kooikerhondje	1.5%
English Cocker Spaniel	1.3%
Pyrenean Sheepdog	1.3%
Finnish Lapphund	1.3%
Kromfohrländer	1.2%
Swedish Vallhund	1.2%
Kerry Terrier	1.1%
German Miniature Spitz	1.1%
Lapponian Herder	1.1%
Mudi	1.1%
Schapendoes	1.1%
Danish–Swedish Farmdog	1.1%
Miniature Schnauzer	0.9%
Papillon	0.9%
German Medium Spitz	0.8%
Pumi	0.8%
German Shepherd	0.7%
Schipperke	0.7%
Bichon Frise	0.7%
Black and Tan English Toy Terrier	0.5%
Lagotto Romagnolo	0.5%
Lancashire Heeler	0.5%
Staffordshire Bull Terrier	0.5%
Cavalier King Charles Spaniel	0.5%
Chihuahua	0.4%
Standard Poodle	0.4%
Japanese Spitz	0.4%
Standard Schnauzer	0.4%
Toy Poodle	0.4%
Wales Springer Spaniel	0.4%
Whippet	0.4%

Boston Terrier	0.3%
Breton	0.3%
Coton de Tuléar	0.3%
Dalmatian	0.3%
Havanese	0.3%
Icelandic Sheepdog	0.3%
Keeshond	0.3%
Chinese Crested	0.3%
Manchester Terrier	0.3%
Nova Scotia Duck Tolling Retriever	0.3%
Phalène	0.3%
Polish Lowland Sheepdog	0.3%
Bohemian Shepherd Dog	0.3%
Wheaten Terrier	0.3%
Wales Terrier	0.3%
Welsh Corgi Cardigan	0.3%
Alaskan Malamute	0.1%
American Hairless Terrier	0.1%
American Staffordshire Terrier	0.1%
Appenzeller Sennenhund	0.1%
Beagle	0.1%
Bearded Collie	0.1%
Beauceron	0.1%
Brazilian Terrier	0.1%
Croatian Sheepdog	0.1%
Dutch Shepherd	0.1%
English Springer Spaniel	0.1%
German Hunting Terrier	0.1%
German Pinscher	0.1%
Giant Schnauzer	0.1%
Griffon Bruxellois	0.1%
Golden Retriever	0.1%
Gordon Setter	0.1%
Hovawart	0.1%
Lhasa Apso	0.1%
Löwchen	0.1%
Pomerian	0.1%
Portuguese Podengo	0.1%
Prague Ratter	0.1%
Silk Terrier	0.1%
Tibetan Spaniel	0.1%
Volpino Italiano	0.1%
